# Effective temperature management

**DOI:** 10.1186/1471-227X-15-S1-A13

**Published:** 2015-06-24

**Authors:** Ian Tweedie

**Affiliations:** 1 Department of Neuroanaesthesia and Critical Care, The Walton Centre NHS FT, Liverpool, UK

## 

‘It is not enough just to influence body temperature – you have to control it’ (M Holzer, Therapeutic Temperature Management (TTM) 2012, Berlin). For this to be possible we need the ideal cooling equipment/technique and a reliable temperature monitoring technique. Many techniques are available [1], but only two types meet most of the ideal characteristics – automatic temperature feedback-controlled surface hydrogel pads or endovascular cooling devices.

To be fully effective, control is needed for all three phases of TTM.

Induction – the how and when are to an extent disease dependent. The evidence is mainly animal based [2] and the human evidence can be conflicting [3], although the suggestion appears to be the sooner the better. Decision to target temperature times are similar for both types of devices, but may be enhanced with the use of adjuncts, such as intravenous infusion of cold fluids. Surface cooling is negatively influenced by obesity and pyrexia, with 32% of patients failing to reach the target in <12 hours [4].

Maintenance – how deep and how long? Some guidance does exist for depth on a disease process basis (for example, 2010 European resuscitation guidelines). Most evidence is available for post-cardiac arrest, cooling to <34°C. However, recent evidence suggests that preventing fever by cooling to 36°C is just as effective as cooling to 33°C [5]. Duration appears to correlate with outcome. Animal studies support this [2] and human studies are suggestive. For TBI, mortality is definitely better but neurologic outcome is less clear [6]. Although each technique has its own advantages, overall both seem equally effective at maintaining a stable target temperature [7]. However, for it to be successful, complications of TTM need to be effectively managed.

Rewarming – often the Cinderella, but it has been long known that it can affect the neurological outcome. The question that has as yet to be answered is how fast? The consensus based on evidence available is that slow is best for the brain for both mortality and neurological outcome [6], whilst avoiding post-rewarming fever. Both types of device seem to be able to control rewarming adequately.

So how can you choose which device to use, as each seems to be effective in all three phases? Long-term use of endovascular devices for fever control is associated with a higher incidence of VTE and PE [8]. Thus, for longer term temperature management the surface device may be the more appropriate

In conclusion, effective temperature control is essential for TTM, this is best achieved with temperature feedback control devices, and management of complications is an essential part of the care. Is fever control the new goal for post-cardiac arrest and ischaemic stroke?

## Financial disclosure

IT has received an honorarium and travel costs from C. R. BARD.

## Declaration

This abstract and supplement was proposed, developed and commissioned by BMC Emergency Medicine and was funded by an educational grant from C. R. BARD, NJ, USA. The published abstract was independently prepared by the author. C. R. BARD had no input into the content.
